# Harnessing plant–microbe interactions: strategies for enhancing resilience and nutrient acquisition for sustainable agriculture

**DOI:** 10.3389/fpls.2025.1503730

**Published:** 2025-04-15

**Authors:** Abdulhamid Yusuf, Min Li, Si-Yu Zhang, Fidelis Odedishemi-Ajibade, Rui-Fang Luo, Ya-Xiao Wu, Ting-Ting Zhang, Adamu Yunusa Ugya, Yunzeng Zhang, Shuo Duan

**Affiliations:** ^1^ Jiangxi Provincial Key Laboratory of Pest and Disease Control of Featured Horticultural Plants, Gannan Normal University, Ganzhou, Jiangxi, China; ^2^ Joint International Research Laboratory of Agriculture and Agri-Product Safety of the Ministry of Education, Yangzhou University, Yangzhou, Jiangsu, China; ^3^ Department of Plant Science and Biotechnology, Federal University, Dutsin-ma, Katsina State, Nigeria; ^4^ Department of Civil and Environmental Engineering, Federal University of Technology Akure, Akure, Nigeria; ^5^ Department of Environmental Management, Kaduna State University, Kaduna State, Kaduna, Nigeria

**Keywords:** rhizobiome engineering, plant-microbe interactions, synthetic microbial communities (SynComs), root exudates, sustainable agriculture, soil health

## Abstract

The rhizosphere, a biologically active zone where plant roots interface with soil, plays a crucial role in enhancing plant health, resilience, and stress tolerance. As a key component in achieving Sustainable Development Goal 2, the rhizosphere is increasingly recognized for its potential to promote sustainable agricultural productivity. Engineering the rhizosphere microbiome is emerging as an innovative strategy to foster plant growth, improve stress adaptation, and restore soil health while mitigating the detrimental effects of conventional farming practices. This review synthesizes recent advancements in omics technologies, sequencing tools, and synthetic microbial communities (SynComs), which have provided insights into the complex interactions between plants and microbes. We examine the role of root exudates, composed of organic acids, amino acids, sugars, and secondary metabolites, as biochemical cues that shape beneficial microbial communities in the rhizosphere. The review further explores how advanced omics techniques like metagenomics and metabolomics are employed to elucidate the mechanisms by which root exudates influence microbial communities and plant health. Tailored SynComs have shown promising potential in enhancing plant resilience against both abiotic stresses (e.g., drought and salinity) and biotic challenges (e.g., pathogens and pests). Integration of these microbiomes with optimized root exudate profiles has been shown to improve nutrient cycling, suppress diseases, and alleviate environmental stresses, thus contributing to more sustainable agricultural practices. By leveraging multi-disciplinary approaches and optimizing root exudate profiles, ecological engineering of plant-microbiome interactions presents a sustainable pathway for boosting crop productivity. This approach also aids in managing soil-borne diseases, reducing chemical input dependency, and aligning with Sustainable Development Goals aimed at global food security and ecological sustainability. The ongoing research into rhizosphere microbiome engineering offers significant promise for ensuring long-term agricultural productivity while preserving soil and plant health for future generations.

## 1 Introduction

The incessant increase in population growth and shift in dietary preferences have led to a greater demand for food production and distribution worldwide. This has put pressure on agricultural systems and resources, leading to concerns about sustainability and food security in the future. Several agricultural solutions and practices for maintaining food security and environmental sustainability are facing setbacks. For example, conventional agricultural practices, which rely heavily on synthetic fertilizers and pesticides, have been shown to have detrimental impacts on soil health, biodiversity, and water resources (Food and Agriculture Organization of the United Nations (FAO), 2017). Diverse studies have shown that the excessive use of nitrogen fertilizers contributes to soil acidification and water pollution, leading to the eutrophication of aquatic ecosystems (Ahmad et al., 2024). Furthermore, the prevailing unsustainable agricultural model is exacerbated by climate change, resulting in increased abiotic stresses, such as drought and salinity, which significantly hinder crop productivity (Melillo et al., 2017; Eswar et al., 2021). Despite these challenges, there is considerable potential for sustainable agriculture through the utilization of the plant microbiome. This intricate network of microorganisms residing in and around plant roots plays a pivotal role in enhancing plant health, growth, and resilience (Sudheer et al., 2020). These beneficial microbes can improve nutrient uptake, enhance stress tolerance, and promote plant growth by producing phytohormones and other metabolites that support plant development (Sudheer et al., 2020; Eswar et al., 2021). As such, leveraging the plant microbiome represents a promising strategy for developing sustainable agricultural practices that address both food security and environmental challenges.

Recent advances in our understanding of plant–microbe interactions, fueled by cutting-edge next-generation sequencing technologies, have revealed the profound impact of the microbiome on plant performance (Sudheer et al., 2020). Root exudates, a complex mixture of organic compounds released by plant roots, act as a vital communication channel between plants and their microbial partners. This exudate composition, encompassing sugars, amino acids, organic acids, and secondary metabolites, shapes the composition and functionality of the rhizosphere microbiome. By serving as a nutrient source for microorganisms, root exudates influence microbial growth and diversity, ultimately having beneficial effects on the plant. Understanding the intricate interplay between root exudates and the rhizosphere microbiome opens exciting avenues for manipulating microbial communities to enhance crop productivity (Kuroyanagi et al., 2022).

One promising strategy is the development of synthetic microbial communities carefully curated consortia of microorganisms tailored to perform specific functions (Dolinsek et al., 2016). These synthetic communities, designed for specific plant species and environmental conditions, hold the potential to address the limitations of traditional single-strain inoculants (Alahmad et al., 2024). Constructing effective synthetic communities necessitates a comprehensive understanding of the complex interactions within the rhizosphere microbiome, including identifying key microbial players, deciphering their functional roles, and elucidating how these interactions are influenced by root exudates. By carefully selecting and combining microorganisms with complementary functions, synthetic communities can be designed to promote plant growth, enhance nutrient uptake, and bolster resilience against various abiotic and biotic stresses (Timofeeva et al., 2023; Tran et al., 2024). For example, synthetic communities can be engineered to enhance nitrogen fixation, a crucial process for plant growth, by combining nitrogen-fixing bacteria with microorganisms that facilitate the availability of essential nutrients like phosphorus. Similarly, synthetic communities can be developed to improve drought tolerance by integrating microorganisms that enhance water uptake, promote root growth, or produce plant growth-promoting hormones (Timofeeva et al., 2023; Santander et al., 2024). This multidisciplinary endeavor requires the integration of knowledge from microbiology, plant science, and ecology. Future research should investigate the intricate interactions within the rhizosphere microbiome, the influence of root exudates, and the specific needs of different plant species and environmental contexts (More et al., 2014; Pantigoso et al., 2022a).

The application of microbiological inoculants presents a substantial opportunity to improve agricultural landscapes. Nonetheless, there is a concerning dearth of in-depth exploration regarding the mechanisms driving these interactions, particularly the critical interplay between plants and microbes in the rhizosphere. Understanding these dynamics is essential for maximizing the potential of agricultural practices and ensuring sustainable land management (Liu et al., 2014; O’Callaghan et al., 2022; Astapati and Nath, 2023). Scholars have significantly contributed to advancing the development and engineering of microorganisms, leading to the creation of biological pesticides, herbicides, control agents, and fertilizers. These beneficial inoculants help minimize the impact of chemical inputs while enhancing both the quality and quantity of agricultural products (Barrera-Galicia et al., 2021; Astapati and Nath, 2023).

This comprehensive review seeks to examine the intricate relationship between root exudates and the rhizosphere microbiome, emphasizing the mechanisms by which these interactions influence plant health and productivity through an extensive literature search. The goal is to leverage the functionalities of the microbiome to enhance crop production and deliver safe, high-nutrition food products. Understanding the interconnectedness of microbiomes in specific environments, their contributions to Sustainable Development Goal (SDG) 2, and their roles in implementing the One Health concept is paramount. Harnessing plant microbiomes for sustainable crop production systems is a critical pursuit.

Moreover, this review explores the potential of SynComs as a powerful tool for sustainable agriculture while shedding light on the challenges and opportunities associated with their development and application. Furthermore, it outlines future research directions in this dynamic field, emphasizing the necessity for a holistic approach that considers the complex interactions within the rhizosphere microbiome and the invaluable role of root exudates. By harnessing the chemical cues of root exudates, this review aspires to pave the way for engineering resilient rhizosphere microbiomes, thereby promoting plant growth and resilience against environmental stresses, ultimately contributing to sustainable and productive agricultural systems.

## 2 Root exudates: biochemical signals for rhizosphere communication

### 2.1 Root exudates and their role in plant–microbe interactions

Root exudates play a pivotal role in shaping the rhizosphere microbiome, functioning as biochemical signals that mediate communication between plants and microorganisms (Figueroa-López et al., 2016; Berg et al., 2020; Poupin et al., 2023; Hacquard and Martin, 2024). These exudates comprise a diverse range of compounds, including sugars, amino acids, organic acids, and secondary metabolites, each serving a specific function in plant–microbe interactions. Studies have shown that root exudates serve as crucial signaling molecules, influencing microbial community composition and functionality by attracting beneficial microbes and deterring pathogens (Solomon and Janda, 2024a). Plants strategically release specific exudates to attract beneficial microorganisms such as nitrogen-fixing bacteria and mycorrhizal fungi, which enhance nutrient acquisition. For instance, legumes release flavonoids that attract nitrogen-fixing bacteria, thereby improving nitrogen availability for plant growth (Tian et al., 2010; Jacoby et al., 2020a; Marín et al., 2021; Chen and Liu, 2024; Wang et al., 2015). Beyond nutrient acquisition, root exudates are critical for pathogen defense and stress tolerance. Under stress conditions such as drought or salinity, plants release targeted exudates that recruit microbes with stress-alleviating properties. These microbes assist plants by improving water uptake, tolerating salinity, or producing growth-promoting hormones (Cao et al., 2024; El-Saadony et al., 2024).

**Figure 1:**
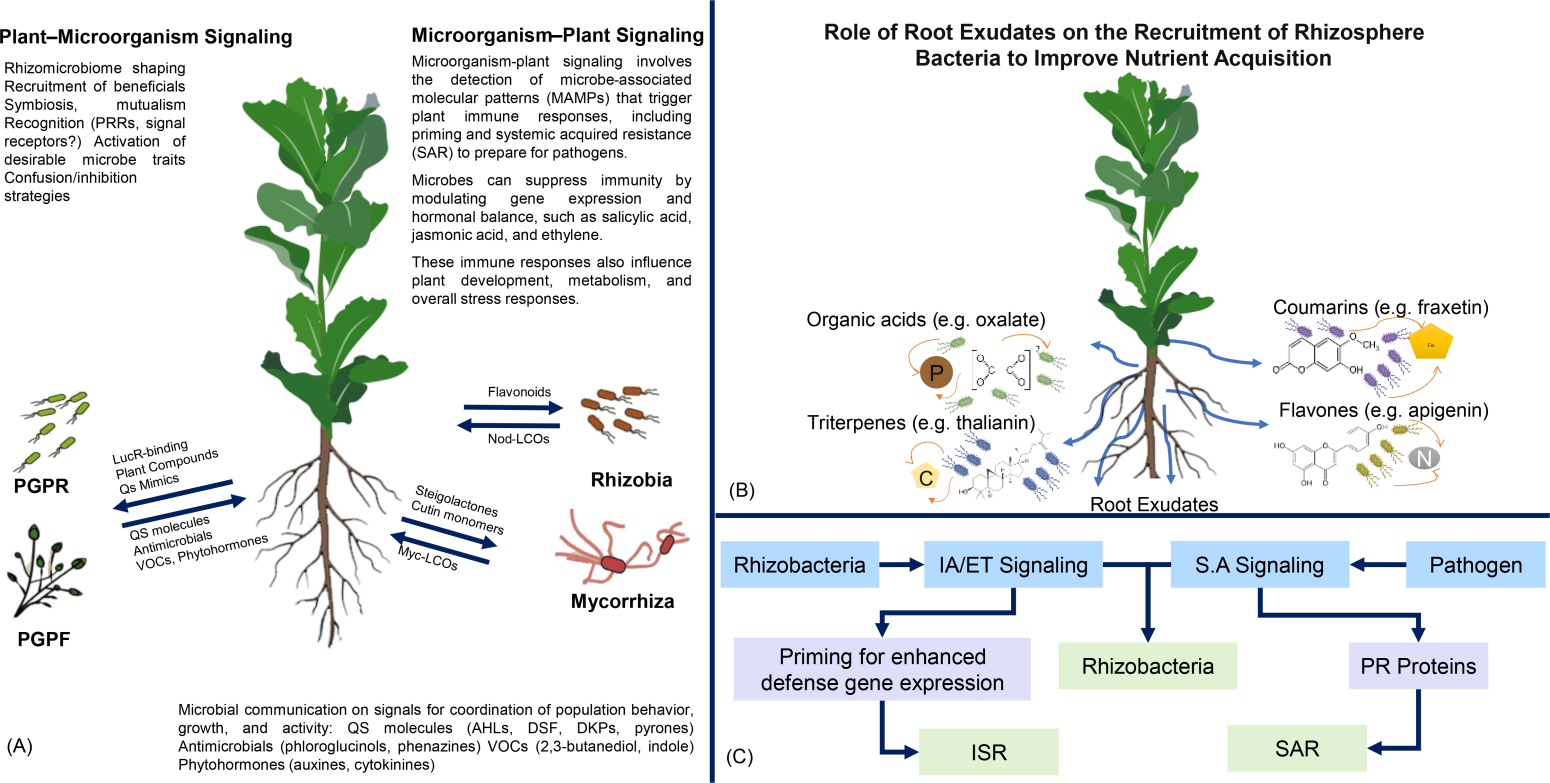
Plant–microorganism interactions and root exudate functionality. This figure illustrates the complex signaling pathways between plants and microorganisms. Panel **(A)** highlights the mechanisms of plant–microorganism signaling, including the recognition of microbe-associated molecular patterns (MAMPs) and the roles of various signaling molecules in plant growth-promoting rhizobacteria (PGPR). Panels **(B, C)** focus on how root exudates facilitate the recruitment of rhizosphere bacteria to enhance nutrient acquisition. It also emphasizes the dual roles of root exudates in priming plant defenses against pathogens and inducing systemic acquired resistance (SAR) through interspecies signaling among microorganisms.

Moreover, root exudates play a significant role in plant growth and development. They influence root architecture by promoting root branching, which increases the surface area available for nutrient and water uptake. Root exudates can also stimulate rhizosphere microbes capable of degrading pollutants, thereby contributing to soil bioremediation (Barreiro and Dί́az-Raviña, 2021; Saeed et al., 2021). **Figure 1** illustrates the multifaceted roles of root exudates in plant–microorganism interactions, highlighting their importance in nutrient acquisition, stress mitigation, and soil health. The chemical composition of root exudates is highly complex and varies depending on plant species, developmental stages, and environmental conditions. The interaction between exudates and the diverse microbial community in the rhizosphere creates a dynamic system (Kumar and Pandey, 2013; Stringlis et al., 2018a; Lombardo et al., 2024). Understanding these interactions offers promising possibilities for sustainable agriculture, allowing for the manipulation of root exudates to enhance nutrient acquisition, improve stress tolerance, and boost plant defenses (Haney, 2021a). As research into the rhizosphere’s chemical language progresses, we gain insights into how plant–microbe interactions can improve crop productivity, soil health, and environmental sustainability (Pantigoso et al., 2022b).

Root exudates not only are important for attracting beneficial microbes but also play a role in regulating microbial populations. They can either attract or repel specific microorganisms, influencing the composition and behavior of the rhizosphere microbiome (Letters, 2014; Hardoim et al., 2015a; Rendueles and Ghigo, 2015; Teixeira et al., 2019; Siddique et al., 2022). For example, sucrose, a disaccharide secreted by plant roots, is involved in the colonization of beneficial gram-positive bacteria, such as *Bacillus* strains. This sugar triggers signaling pathways that enhance bacterial motility and biofilm formation, facilitating root colonization (Richter et al., 2023). Additionally, glucose, a chemoattractant for many fungal strains, also plays a role in bacterial colonization in the maize rhizosphere (Barrera-Galicia et al., 2021; Pantigoso et al., 2022b). Other sugar alcohols, like inositol, promote bacterial growth and processes such as chemotaxis, biofilm formation, and siderophore production (Hardoim et al., 2015a; Siddique et al., 2022; Richter et al., 2023). Flavonoids play an essential role in plant interactions with nitrogen-fixing bacteria. These compounds are structurally diverse and influence the composition of the microbiome in crops like *Arabidopsis*, rice, and maize. Flavonoids promote the growth of beneficial microbes and contribute to biological nitrogen fixation by encouraging the growth of *Gluconacetobacter diazotrophicus* (Liu et al., 2014; Wang X. et al., 2024). Phenolic compounds released by plants act as both defense mechanisms against pathogens and carbon sources for microbes. Coumarins, a type of phenolic secondary metabolite, show selective antimicrobial effects and can inhibit the growth of soil-borne pathogens while promoting beneficial microbes (Rosier et al., 2016; Xu and Dodd, 2022). Moreover, root volatiles [volatile organic compounds (VOCs)] are crucial for long-distance interactions in the soil. These compounds serve as signaling molecules or energy sources for microbes. The composition and concentration of VOCs can vary depending on microbial presence, influencing bacterial growth and competition (Marín et al., 2021). Some VOCs, such as terpenes, increase during pathogen infection, inhibiting pathogen growth while attracting beneficial microbes. Under nutrient limitation, VOCs can serve as info-chemicals, providing cues to beneficial bacteria in the rhizosphere (Stringlis et al., 2018a; Lombardo et al., 2024; Luo et al., 2024). Secondary metabolites like camalexin and benzoxazinoids, which are found in root exudates, help defend plants against pathogens and enrich the soil microbial community (Richter et al., 2023; Xu and Wang, 2023).

Root exudates are essential components in the chemical communication between plants and the rhizosphere microbiome (Zhang et al., 2023). These compounds act as energy sources, nutritional provisions, and signaling molecules that influence microbial activity (Chowdhury et al., 2011). The composition and quantity of root exudates vary with environmental conditions and plant growth stages, helping plants adapt to changes in nutrient availability, water stress, and pathogen presence. For instance, plants under nutrient stress may release organic acids to acquire scarce nutrients, while drought stress triggers the release of specific exudates to recruit microbes that aid in water retention and stress mitigation (Haney, 2021a; Pantigoso et al., 2022b; Richter et al., 2023).

### 2.2 Factors influencing the abundance and composition of root exudates

Root exudates play a crucial role in shaping the rhizosphere, with their abundance and composition influenced by a variety of biotic and abiotic factors. Plant species and their genetic makeup are key determinants, as different species release distinct exudates to interact with specific microbes in the soil. For example, legumes release flavonoids to attract nitrogen-fixing bacteria, while grasses exude compounds that promote mycorrhizal fungal growth (Solomon and Janda, 2024a). Within a species, varieties and cultivars can also show variation in exudate profiles, impacting microbial interactions (Saeed et al., 2021). The developmental stage of the plant is another important factor. Seedlings generally release more sugars to support their rapid growth, while mature plants may shift to producing organic acids or secondary metabolites for nutrient acquisition and defense against pathogens (Teixeira et al., 2019). Environmental conditions, such as nutrient availability, water stress, and temperature, significantly affect exudate production. For example, nutrient-deficient plants may secrete more organic acids to enhance nutrient uptake, while drought-stressed plants may release specific exudates that attract microbes capable of mitigating water stress (Boot et al., 2013; Rosier et al., 2016; Canarini et al., 2017; Wu et al., 2022). Additionally, the presence of pathogens or beneficial microbes can trigger the release of specific compounds; pathogens may prompt the production of antimicrobial secondary metabolites, while beneficial microbes may stimulate exudates that enhance their growth (Ma et al., 2023). Environmental stressors like biotic (e.g., pathogen attack) and abiotic stress (e.g., soil salinity and drought) also shape the exudate composition, as plants release metabolites to counteract these challenges (Al-Khayri et al., 2023). Root system structure and age influence exudate dynamics, with plants of different root architectures potentially releasing different profiles of exudates and older roots typically releasing fewer exudates than younger ones (Ecevit et al., 2022). Soil characteristics, such as soil type and pH, also play a role in determining exudate composition, with plants in acidic soils potentially releasing more organic acids to solubilize nutrients (Hallama, 2019). Moreover, the microbial community in the rhizosphere can influence the exudate profile, as beneficial microbes may stimulate plants to release exudates that encourage their colonization, while pathogens may prompt defense-related exudates (60). Ultimately, the composition and quantity of root exudates are dynamic and depend on multiple factors, including plant species, environmental conditions, and microbial interactions, making them critical to plant–microbe communication and essential for plant growth, health, and sustainable agriculture (de Menezes et al., 2017; Jiang et al., 2021b).

Some studies indicate that root exudates attract beneficial microorganisms, influence the rhizosphere microbiome, and enhance plant performance (Vogel and Bai, 2016). Plants typically release 11%–40% of their photosynthetic yield into the rhizosphere as root exudates, which can be classified into low- or high-molecular-weight compounds. Low-molecular-weight compounds include a variety of molecules such as sugars, organic acids, amino acids, alcohols, volatile compounds, and other secondary metabolites (Lin et al., 2021; Poupin et al., 2023). High-molecular-weight compounds comprise mucilage (polysaccharides) and proteins, which tend to exhibit reduced variability while typically constituting a larger proportion of total exudates. Low-molecular-weight compounds, including sugars and amino acids, are primarily released through passive mechanisms such as diffusion, channel transport, and vesicular transport (Hardoim et al., 2015a; Wang et al., 2020). The export of sugars is facilitated by “sugars will eventually be exported transporters” (SWEETs) that function along a concentration gradient (Jiang et al., 2021b). Other compounds, such as secondary metabolites, proteins, and polysaccharides, are typically secreted into the rhizosphere via active mechanisms involving various membrane-bound proteins. For example, secondary metabolites—including flavonoids, phenolics, and hormones—are actively secreted into the rhizosphere through ATP-binding cassette (ABC) transporters or multidrug and toxic compound extrusion (MATE) transporters. Citrate, a low-molecular-weight compound, is specifically transported by MATE antiporters (Lin et al., 2021). Overall, the composition of root exudates and the mechanisms of their release are influenced by various factors including plant species, developmental stages, environmental conditions, and interactions with soil microorganisms, which collectively shape the rhizosphere dynamics (De Vleesschauwer et al., 2008).

### 2.3 Root-derived metabolites for plant–microbe communication

Root exudates contain various substances that serve as energy sources, nutrient supplies, and communication signals for plants and rhizosphere microorganisms, influencing the attraction or repulsion of specific microbial strains (Xu and Dodd, 2022; Boo et al., 2024). This study will examine specific compounds, including organic acids, amino acids, sugars, sugar alcohols, flavonoids, phenolic compounds, volatiles, and other secondary metabolites (see **Table 1**). These compounds function as nutrients, signals, or antimicrobial agents in the colonization process of beneficial soil rhizobacteria. For example, various organic acids, including malic, citric, fumaric, tartaric, and succinic acids, are essential nutrients and signaling molecules for beneficial soil rhizobacteria during the colonization process (De Vleesschauwer et al., 2008). Treatments with these acids have been shown to enhance soil physicochemical conditions and manipulate microbial community dynamics, promoting the growth and colonization of beneficial bacteria via mechanisms such as chemotaxis, bacterial motility, and biofilm formation (Stassen et al., 2021; Richter et al., 2023). For example, fumaric and tartaric acids positively influence biofilm formation in *Hansschlegelia zhihuaiae* by regulating proteins related to motility, which facilitates the degradation of certain herbicides. Additionally, oxalate serves as a carbon source specifically for beneficial *Burkholderia* species, while pathogenic strains are unable to utilize it, highlighting the selective roles of organic acids in rhizosphere dynamics (Xu et al., 2020; Barrera-Galicia et al., 2021; Coban et al., 2022).

**Table 1:** Root-derived metabolites as signals and substrates for beneficial bacteria.

Root-derived metabolite	Examples	Signaling or substrate functions	References
Coumarin	Scopoletin	Iron deficiency, recruitment of iron-reducing bacteria	(Patzner et al., 2020; Stassen et al., 2021)
Isoflavonoids	Daidzein, genistein	Regulation of nodule factors	(Wu et al., 2024)
Benzoxazinoids	Indole-3-glycerol-phosphate	Trigger colonization of plant growth-promoting rhizobacteria	(Zhang et al., 2017)
Phytoalexin	Camalexin	Stimulate plant growth-promoting rhizobacteria	(Eswar et al., 2021)
Flavones	Apigenin, luteolin	Enrich Oxalobacteraceae under nitrogen deprivation	(Kumar and Pandey, 2013; Medina and Kutuzova, 2022)
Triterpenes	Thalainin, thalianyl	Carbon source for bacterial proliferation	(Zhou et al., 2012; Hardoim et al., 2015a)
Fatty acids	2-Methylbutyric, palmitic acid	Enhance bacterial recruitment under salinity stress	(Marín et al., 2021)
Amino acids	Tryptophan, histidine, arginine	Chemotaxis to plant growth-promoting bacteria	(Chen and Liu, 2024)
Sugars	Sucrose, polysaccharides, glucose	Motility, colonization, biofilm formation signals	(Richter et al., 2023)
Sugar alcohols	Inositol	Flagellar development, chemotaxis, biofilm formation	(Peters et al., 2010; Xue et al., 2021)
Volatiles	Terpenes, ketones	Act as nutrients, info-chemicals for chemotaxis	(Lin et al., 2021)
Phenolics	Fraxetin, scopoletin	Selective antimicrobial effects on microbial strains	(Ecevit et al., 2022)

Amino acids are critical components of root exudates that provide essential nitrogen sources for soil microbes, thereby stimulating the growth of beneficial rhizobacteria. Specific amino acids selectively enhance beneficial over pathogenic bacterial growth; for instance, histidine and aspartate are crucial in mediating the chemotactic response of *Azorhizobium caulinodans* (Kleber et al., 2015; Wu et al., 2024). However, amino acids can also support the proliferation of pathogenic bacteria, underscoring a complex interplay within microbial communities (Zhang et al., 2017). Sugars, particularly sucrose, serve as significant carbon sources and signaling molecules for rhizobacteria, influencing colonization dynamics. Sucrose triggers signaling cascades that activate motility in bacteria such as *Bacillus subtilis*, thus facilitating effective root colonization (Kumar and Pandey, 2013). Furthermore, sucrose induces the synthesis of antibiotics like surfactin, which enhances the competitive colonization efficacy of *Bacillus*. Other plant polysaccharides also contribute to biofilm formation and promote flagellar activity in bacteria, supporting robust colonization (Liu et al., 2014; Richter et al., 2023; Li et al., 2024).

Sugar alcohols, such as inositol, are significant in root exudates, where they function as both carbon sources and signaling molecules. Inositol promotes several microbiological processes, including bacterial chemotaxis and biofilm formation (Medina and Kutuzova, 2022). Its transport and secretion in plants are regulated by specific transporters, and its metabolism can boost bacterial colonization competence, emphasizing its functional significance in the rhizosphere (Nazir et al., 2014).

Flavonoids play a vital role in plant–bacteria interactions, especially nitrogen-fixing species (Yang et al., 2018). They attract specific microbial strains by modulating gene expression relevant to motility and biofilm production. Certain flavonoids have been associated with stress resilience in plants and the promotion of biological nitrogen fixation, illustrating the intricate relationships within plant–microbe dynamics (Kumar and Pandey, 2013; Xue et al., 2021). Phenolic compounds released by plants serve as signaling molecules and antimicrobial agents, attracting beneficial microbes while inhibiting the growth of pathogens through various mechanisms, thus enhancing plant defense mechanisms and favorably altering microbial community structures (Pierce et al., 2020). Root volatiles, including various VOCs, facilitate long-distance interactions within the rhizosphere and serve as signals regulating microbial growth and competition. VOCs can effectively attract beneficial bacteria while deterring pathogens, demonstrating diverse effects on microbial community composition that depend on environmental conditions (Li et al., 2024). Additionally, secondary metabolites in root exudates such as camalexin and benzoxazinoids are significant in mediating plant–microbe interactions. These compounds enhance the growth of beneficial microbial strains and contribute to plant defense against pathogens, thereby influencing the overall composition of the rhizosphere microbiome (Wang Y. et al., 2023).

In conclusion, the variety of compounds present in root exudates including organic acids, amino acids, sugars, flavonoids, and secondary metabolites plays crucial roles in shaping microbial communities and enhancing plant health. These complex interactions are fundamental for maintaining healthy soil ecosystems and sustainable agricultural practices.

## 3 Enhancing plant–microbe interactions

### 3.1 The impact of rhizosphere microbiome on plant growth, resilience, and sustainable agriculture

The rhizosphere, the soil region directly influenced by plant roots, is a dynamic ecosystem teeming with microbial organisms that play a crucial role in plant growth and resilience. Effective management of this microbial community holds immense potential for improving plant adaptability and achieving sustainable agricultural practices (He et al., 2010; Saeed et al., 2021; Medina and Kutuzova, 2022). **Table 2** summarizes a variety of microbes and crops, illustrating how rhizosphere engineering can harness indigenous plant microbes and foster beneficial plant–microbe interactions (Yang et al., 2018). The concept of the “core” microbiome is pivotal for understanding the microbial communities associated with plants. This core microbiome consists of a unique set of microbes specific to each plant species, which significantly influences plant health and development (Rendueles and Ghigo, 2015; Risely, 2020). These microbes are often inherited from parent plants, while the plant’s unique root exudates and signaling mechanisms further shape this microbiome. The plant microbiome encompasses a diverse and complex community of microorganisms that inhabit various plant structures, including roots, stems, leaves, flowers, and seeds. These microbial communities are integral to plant development, recovery, and overall health (Zu et al., 2012; Astapati and Nath, 2023).

**Table 2:** Diversity of microbes and host plants highlighting their potential for rhizosphere engineering.

Rhizo-microorganisms	Host plant	Microorganism type	Signaling pathways	Reference
*Pseudomonas fluorescens*	*Oryza sativa*	Bacteria	Abscisic acid	(De Vleesschauwer et al., 2008)
*Glomus versiforme* (AMF), *Bacillus methylotrophicus*	*Nicotiana tabacum*	Fungi, bacteria	Abscisic acid, indole acetic acid	(Lindahl and Olsson, 2014; Liu et al., 2014; Wu et al., 2024)
*Brevibacterium linens*	*Eucalyptus grandis*	Bacteria	Ethylene	(Medina and Kutuzova, 2022)
*Bacillus cereus*	*Lycopersicum esculentum*	Bacteria	Ethylene	(Yang et al., 2018)
*Dietzia natronolimnaea*	*Triticum aestivum*	Bacteria	Salt overly sensitive, Abscisic acid	(Berendsen et al., 2018)
*Bacillus* sp., *Enterobacter* sp.	*T. aestivum*, *Zea mays*	Bacteria	Indole acetic acid	(Compant et al., 2025)
*Bacillus* sp., *Pseudomonas* sp.	*T. aestivum*, *Z. mays*	Bacteria	Abscisic acid	(Burlinson et al., 2011)
*Trichoderma asperellum*	*Lolium multiflorum*	Fungi	Indole acetic acid, abscisic acid, gibberellin	
*Bacillus licheniformis*	*Arabidopsis thaliana*	Bacteria	Jasmonic acid, abscisic acid	(Nuccio et al., 2020)
*Burkholderia phytofirmans*	*A. thaliana*	Bacteria	Reactive oxygen species	(Xu et al., 2020; dos Santos et al., 2022)
*Bacillus safensis*, *Ochrobactrum pseudogrignonense*	*T. aestivum*	Bacteria	Reactive oxygen species	(Jiang et al., 2021b)
*Bacillus velezensis*	*T. aestivum*	Bacteria	Reactive oxygen species, abscisic acid	(Zhong et al., 2024)
*Rhodopseudomonas palustris*	*O. sativa*	Bacteria	Abscisic acid, jasmonic acid, ethylene	(Boo et al., 2024)
*Funneliformis mosseae*, *Paraburkholderia graminis*	*Solanum lycopersicum*	Fungi, Bacteria	Reactive oxygen species	(Fan et al., 2017; Wang Y. et al., 2023)
*Enterobacter aerogenes*	*O. sativa*	Bacteria	Indole acetic acid, ethylene	(Notz et al., 2012)
*B. methylotrophicus*, *B. licheniformis*, *Bacillus aryabhattai*	*Spartina maritima*	Bacteria	Cytochrome oxidase, alternative oxidase	(Bilal et al., 2021)
*Paecilomyces formosus*, *Penicillium funiculosum*	*Glycine max*	Fungi	Indole acetic acid, gibberellin	(Bilal et al., 2017)
*Stenotrophomonas maltophilia*, *Agrobacterium fabrum*	*Momordica charantia*	Bacteria	Ethylene	(Starke et al., 2016)

AMF, arbuscular mycorrhizal fungi.

Recent studies have illuminated intricate relationships between plants and their microbial associates. For instance, legumes release flavonoids into the rhizosphere, activating nod genes in rhizobia and leading to the formation of lipo-chito-oligosaccharides (LCOs) recognized by LysM receptor-like kinases (RLKs) in plant roots, which trigger root nodule formation (Hallama, 2019; Xu and Wang, 2023). Similarly, plants interact with arbuscular mycorrhizal fungi (AMF) through strigolactones, promoting the production of Myc factors that are also recognized by LysM RLKs, thus facilitating AMF invasion (Jiang et al., 2021a; Jiang et al., 2021b). These insights are central to developing strategies for engineering the rhizosphere microbiome, ensuring a balanced and resilient ecosystem that supports sustainable agricultural practices (Pierce et al., 2020). Establishing a state of homeostasis in root microbiomes involves understanding the complex interactions between plants and their microbial communities. The concept of fitness plays a vital role in this context, as plants and their microbiomes mutually adapt to environmental conditions, shaping their interactions (Li et al., 2024). The “extended phenotype” theory posits that the rhizomicrobiome is an inherited ecological characteristic of the plant (Wang Y. et al., 2023). However, the movement and composition of microorganisms within the rhizosphere are dynamic, changing with plant development and influencing the microbial community at different growth stages (Xue et al., 2021). These variations underscore the complexity of the rhizosphere and highlight the necessity for detailed studies to optimize it for sustainable agriculture.

### 3.2 Factors affecting the rhizosphere microbiome’s structure and diversity

**Figure 2:**
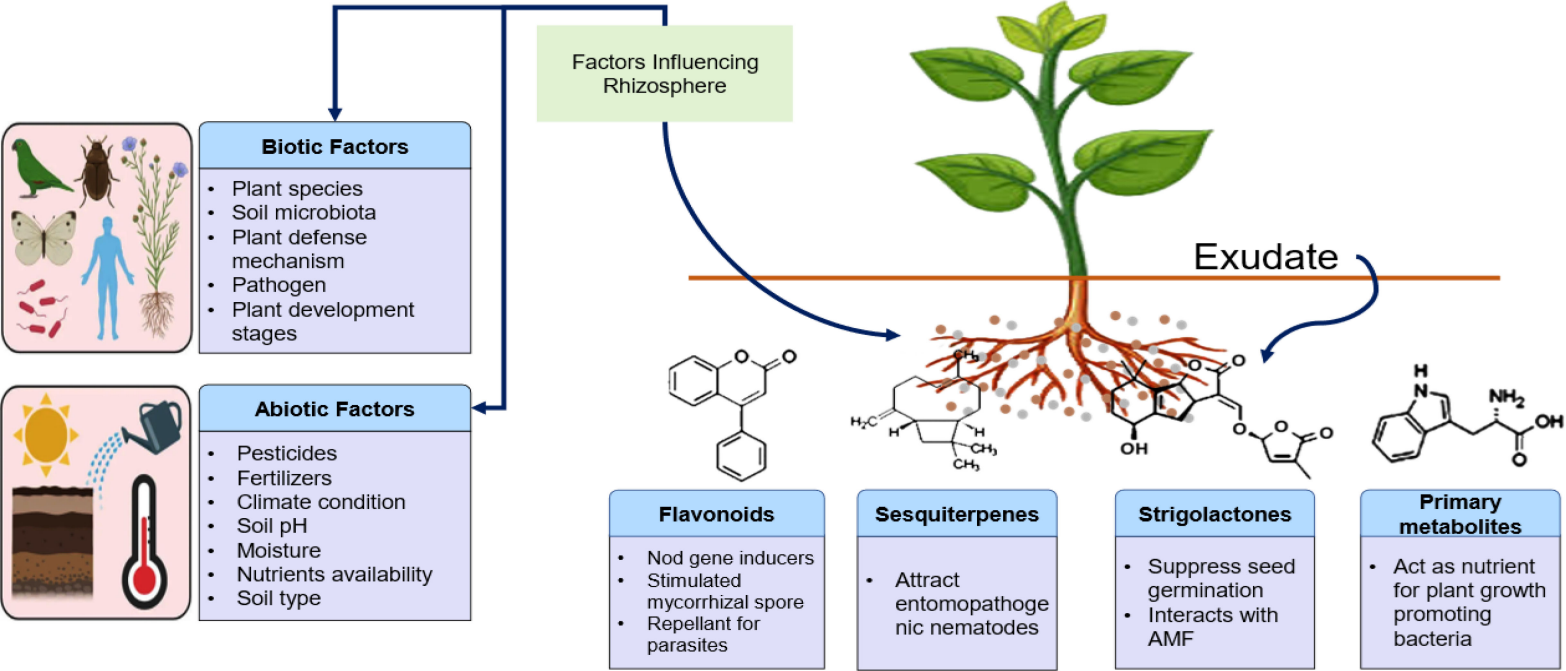
Factors influencing rhizosphere microbiome structure and diversity. The figure illustrates the biotic and abiotic factors that affect the structure and diversity of the rhizosphere microbiome. Biotic factors include plant species, soil microbiota, and plant–pathogen interactions, while abiotic factors encompass elements such as soil pH, moisture, and nutrient availability. Key compounds produced by plants, such as flavonoids, sesquiterpene lactones, and primary metabolites, play significant roles in shaping microbial communities in the rhizosphere.

The structure and diversity of plant microbial communities are shaped by several factors, including the plant’s species, genotype, age, developmental stage, health, and fitness (Jin et al., 2024). The genetic diversity of the host, including its genotype and species, plays a critical role in determining microbial outcomes in the rhizosphere (Vorholt et al., 2017). Studies indicate that different rice cultivars harbor distinct microbial communities, with some varieties showing higher populations of nitrogen-fixing bacteria. Interestingly, the host genome appears to have a greater influence on above-ground microbiota compared to those in the roots or rhizosphere (Franco-Duarte et al., 2019; Compant et al., 2025). Environmental conditions, both biotic and abiotic such as geographic location, soil quality, and climate, also significantly affect microbial composition (Blois et al., 2013; Jansson and Hofmockel, 2020; Itabangi et al., 2022; Astapati and Nath, 2023). These factors (**Figure 2**) primarily influence the rhizosphere and phyllosphere microbiome, but host plant species and genotypes remain crucial for shaping these microbial communities. For example, drought stress has been shown to alter the rhizosphere and phyllosphere microbiome by promoting opportunistic pathogens (Figueroa-López et al., 2016). In addition to abiotic factors, biological factors such as interactions between resident microorganisms, pathogens, soil animals, and weeds also contribute to microbiome dynamics. A study examining microbiome diversity across continents and various plant species showed that soil nutrients and herbivores were key drivers of diversity (Letters, 2014; Butkovi and Gonz, 2021). These findings suggest that universal principles may predict microbial diversity across spatial scales and plant species.

### 3.3 Microbe–microbe interactions as a driver of SynCom outcomes

Microbial interactions within SynComs significantly influence their effectiveness. Studies on extensive SynComs have revealed that while negative interactions are common between different microbial kingdoms (fungi, oomycetes, and bacteria), positive interactions tend to occur within the same kingdom (Richards et al., 2017; Bilal et al., 2021). This has important implications for plant growth and health. For instance, specific microbial groups can either enhance or inhibit plant growth depending on the microbial composition and competition dynamics (**Table 2**). The presence or absence of specific microbial groups can greatly impact plant health and development, highlighting the importance of selecting the right microbial community members. Competition among bacterial strains, particularly within the rhizosphere, plays a crucial role in determining the fitness of nitrogen-fixing rhizobia and other beneficial microbes (López-Mondéjar et al., 2019; dos Santos et al., 2022). Understanding these interactions is key to optimizing SynComs for agricultural applications.

Plants and microbes communicate through specific chemicals, exchanging signals that regulate microbial behavior and plant growth. For example, plants release flavonoids and strigolactones to facilitate beneficial relationships with rhizobia and AMF (Starke et al., 2016; Bilal et al., 2017; Boo et al., 2024). Conversely, microorganisms produce quorum-sensing signal molecules to coordinate their activities, which in turn influence plant growth and immune responses (Zhuang et al., 2020). Legumes produce flavonoids that attract nitrogen-fixing rhizobia, enhancing nitrogen availability for host plants. This interaction exemplifies how plants recognize beneficial microorganisms through specific surface components and released chemicals, known as microorganism-associated molecular patterns (MAMPs) (Lee et al., 2020).

To deepen our understanding of these complex interactions, studies increasingly utilize synthetic microbial community (SynCom)-engineered microbial consortia that mimic natural communities in the rhizosphere. These consortia serve as powerful tools for investigating the dynamic relationships between plants and microbes, particularly in nutrient acquisition and stress response. Studies using SynComs have shown that plant immune signaling pathways are closely linked to microbial community composition. Certain combinations of beneficial microbes can enhance plant health and resilience under stress by modulating immune responses and fostering beneficial interactions (Fall et al., 2022). SynCom, a simplified and controllable microbial community, is an essential tool for validating plant holobiont interactions, identifying core microbiomes, and enhancing crop growth and stress resilience in plant microbiome studies (Hao et al., 2024). Therefore, by systematically varying SynCom composition, insights can be gained into how specific microbial interactions influence plant growth and stress tolerance.

In studies focused on evaluating the traits of plant growth-promoting rhizobacteria (PGPR), smaller SynComs were typically employed, emphasizing functional assessments over ecological analyses. This approach aimed to simplify the incorporation of the “community factor” or interactions responsible for varying outcomes. A top-down study investigated the natural bacterial community within the garlic rhizosphere to identify PGPR strains that positively influenced radish seedlings (*Raphanus sativus*) (Zhuang et al., 2020). Another study reported that greater richness in *Pseudomonas* communities contributes positively to both plant biomass and nutrient levels. Additionally, they revealed that multistrain microbial inoculants promote plant growth more consistently and effectively than individual strain inoculants (Sorensen et al., 2016).

Furthermore, smaller SynComs have been utilized to evaluate community impacts on various PGPR traits, including biocontrol and modulation of host immune responses. Another study examined the microbial community structures in diseased and healthy tomato plants, noting differences in the abundance of Gram-positive Actinobacteria and Firmicutes phyla linked to infection status. They created a SynCom comprising four Gram-positive species that, while not directly antagonistic to the pathogen, induced a heightened immune response. Notably, the duration of plant protection offered by SynCom exceeded that provided by the individual strains (Lee et al., 2020).

SynComs are increasingly being used to study these interactions and their role in shaping microbial communities in the rhizosphere. Studies using SynComs have shown that plant nutritional conditions and the presence of other microbes influence microbial colonization patterns, underscoring the significance of plant immune signaling in microbial community composition (Allison et al., 2014; Lee et al., 2019). Microorganisms play an essential role in improving tolerance to abiotic stresses such as drought, high salt levels, and extreme temperatures. They do this by producing biopolymers, osmolytes, and antioxidant enzymes (Bhardwaj et al., 2013; Thakur et al., 2017; Alster et al., 2018). For instance, *Bacillus* strains have been shown to enhance drought resistance in maize and wheat by stimulating root growth and improving water uptake (Ling et al., 2022; Zhong et al., 2024).

Microbial activities also enhance nutrient absorption by plants. Through nitrogen fixation, nutrient mobilization, and phytohormone synthesis, microorganisms help plants acquire vital nutrients such as nitrogen, phosphorus, and potassium (Egamberdieva et al., 2017). For example, rhizobial inoculants used in legume crops significantly improve nitrogen fixation, reducing the need for synthetic fertilizers. Additionally, microorganisms help plants resist diseases by producing defense enzymes and antimicrobial substances (Pan and Yu, 2011; Blume et al., 2012; Afridi et al., 2024).

#### 3.3.1 Chemical signaling and plant–microbe communication

Plants and microbes communicate through chemical signals that regulate microbial behavior and plant growth. For example, plants release flavonoids and strigolactones to attract and support beneficial microbes like nitrogen-fixing rhizobia and arbuscular mycorrhizal fungi (Kumar and Pandey, 2013; Zeilinger et al., 2016). In turn, microorganisms produce quorum-sensing molecules to coordinate their activities, influencing plant immune responses and growth. This chemical dialogue not only regulates microbial colonization in the rhizosphere but also impacts plant resilience and health (Cotrufo and Lavallee, 2022). Understanding these signaling pathways not only illuminates fundamental biological processes but also offers potential strategies for manipulating microbial communities to improve plant health and productivity. This is particularly crucial in agricultural settings where enhancing crop resilience to abiotic and biotic stresses can lead to sustainable farming practices.

#### 3.3.2 Enhancing plant resilience and nutrient acquisition

**Figure 3:**
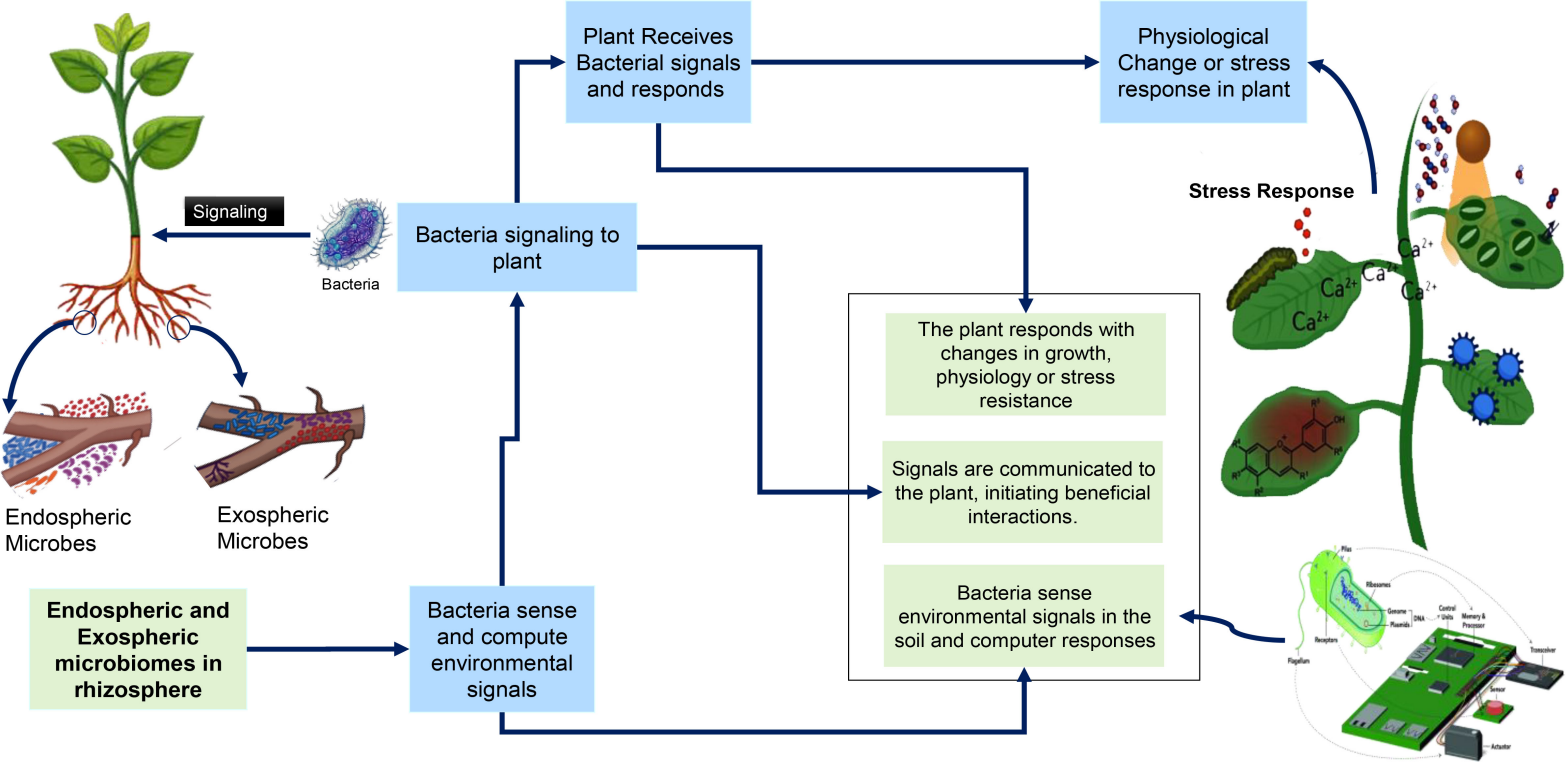
Interactions between root-associated microbes and plant signaling. This figure illustrates the signaling pathways and interactions between endospheric and exospheric microbes in the rhizosphere and their impact on plant responses. It highlights how bacteria sense environmental signals, communicate with plants, and initiate.

Microorganisms play an essential role in enhancing plant resilience to abiotic stresses, such as drought, high salinity, and extreme temperatures (**Table 3**). They achieve this by producing biopolymers, osmolytes, and antioxidant enzymes that help plants tolerate environmental stresses (Intergovernmental Panel on Climate Change (IPCC), 2014; Li et al., 2018; Thakur et al., 2019). For example, certain *Bacillus* strains have been shown to enhance drought resistance in maize and wheat by stimulating root growth and improving water uptake (Sheth et al., 2016). In addition to stress tolerance, microbes improve plant nutrient acquisition. Through processes like nitrogen fixation, nutrient mobilization, and phytohormone synthesis, microorganisms help plants acquire vital nutrients such as nitrogen, phosphorus, and potassium. Rhizobial inoculants, for example, significantly boost nitrogen fixation in legume crops, reducing the need for synthetic fertilizers (Notz et al., 2012). Interactions between root-associated microbes and plant signaling are shown in **Figure 3**. Moreover, microbes contribute to plant disease resistance by producing defense enzymes and antimicrobial substances (Phillips et al., 2011; Mäkelä et al., 2016).

**Table 3:** Key benefits of rhizosphere microorganisms in enhancing plant growth, stress resilience, and soil health.

Benefit	Description	Examples	Reference
Abiotic stress tolerance	Microorganisms alleviate stress caused by drought, salinity, and extreme temperatures by producing biopolymers, osmolytes, and antioxidant enzymes.	*Bacillus* strains improve drought resistance in maize and wheat by stimulating root growth and enhancing water uptake.	(Blagodatskaya and Kuzyakov, 2018; Sabrina et al., 2021; dos Santos et al., 2022)
Nutrient absorption	Microbes enhance the availability and uptake of vital nutrients, including nitrogen, phosphorus, and potassium, through nitrogen fixation and phosphate solubilization.	Rhizobial inoculants improve nitrogen fixation in legumes, reducing dependence on synthetic fertilizers. Phosphate-solubilizing bacteria increase phosphorus availability in crops like wheat.	(Singh and Siddiqui, 2010; Zhang et al., 2017)
Induced systemic resistance (ISR)	Beneficial microorganisms trigger ISR, priming plants’ immune systems to defend against pathogens through enzymatic and secondary metabolite production.	*Trichoderma* species manage fungal infections in tomato and wheat, reducing reliance on fungicides.	(Singh and Siddiqui, 2010)
Biological fertilizers	Microbial inoculants with nitrogen-fixing, phosphate-solubilizing, or potassium-mobilizing capabilities act as natural fertilizers, improving crop productivity.	*Rhizobium* in soybean and *Azospirillum* in cereals significantly enhance yields while reducing fertilizer input.	(Yang et al., 2024)
Disease suppression	Beneficial microbes suppress soil-borne pathogens by producing antibiotics, lytic enzymes, and competing for space and nutrients.	*Pseudomonas fluorescens* suppresses *Fusarium* in wheat; *Bacillus subtilis* inhibits *Rhizoctonia solani* in rice.	(De Vleesschauwer et al., 2008; Hu et al., 2014)
Improved soil fertility	Microorganisms enhance soil organic matter decomposition and nutrient cycling, improving soil structure and fertility.	Mycorrhizal fungi enhance phosphorus cycling in maize; *Bacillus* species improve organic matter decomposition in soils.	(Rousk and Bååth, 2011; Allard et al., 2017; Jin et al., 2024)
Agronomic practices	Agronomic interventions, such as soil amendments, cover cropping, and crop rotations, optimize the rhizosphere for beneficial microbes.	Cover cropping with legumes promotes nitrogen-fixing bacteria; compost amendments improve microbial diversity in degraded soils.	(El-Saadony et al., 2024)
Biocontrol of pathogens	Beneficial microorganisms outcompete pathogens or produce antifungal and antibacterial compounds that suppress plant diseases.	*Bacillus amyloliquefaciens* produces lipopeptides to suppress *Fusarium oxysporum* in tomatoes.	(Dudenhöffer et al., 2016; Fan et al., 2017)

## 4 Harnessing the plant microbiome

### 4.1 Approaches for harnessing the plant microbiome

Harnessing the plant microbiome presents significant potential for sustainable crop production. A thorough understanding of plant–microbe interactions is necessary to develop resilient agricultural systems (Morrison et al., 2016). Enhancing these interactions aids nutrient acquisition, promotes growth, and increases stress resistance. For example, PGPR, such as *Bacillus*, *Pseudomonas*, and *Burkholderia*, contribute through mechanisms like nitrogen fixation, nutrient mobilization, hormone production, and disease suppression (Fierer et al., 2012; dos Santos et al., 2022; Wang Y. et al., 2023). These microbes colonize root zones, improving nutrient uptake and suppressing pathogens. Moreover, PGPR can form synergistic relationships with mycorrhizal fungi, further enhancing nutrient absorption and soil health (Lee et al., 2020; Zhuang et al., 2020).

**Figure 4:**
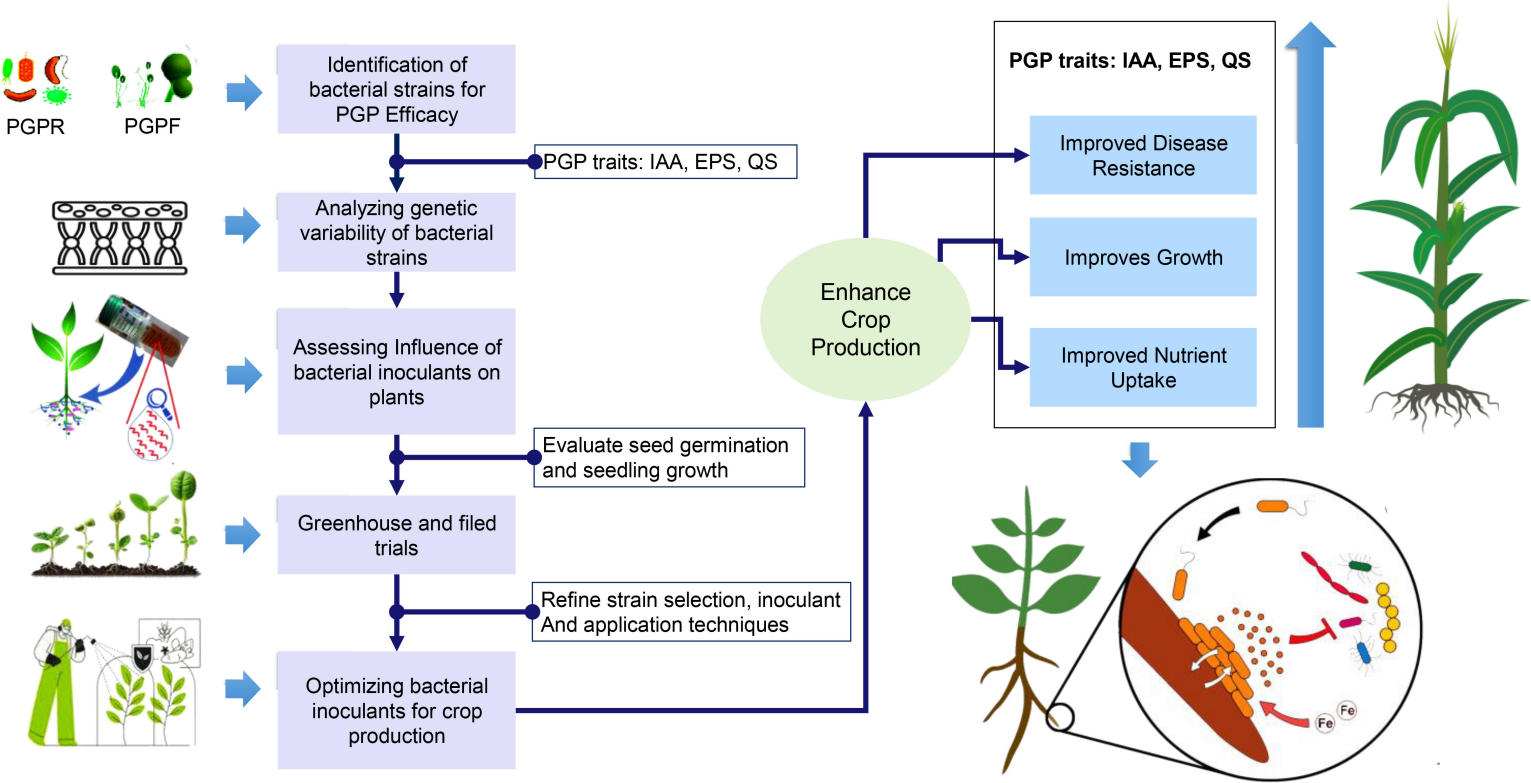
Integrative approach for utilizing bacterial strains as plant growth-promoting inoculant. This figure provides a comprehensive methodology for identifying and optimizing bacterial inoculants with high plant growth-promoting (PGP) efficacy, detailing the steps from screening bacterial isolates for beneficial traits, analyzing genetic variability, assessing the influence of inoculants on plant growth, and refining their application in crop production for enhanced disease resistance, growth improvement, and nutrient uptake.

**Figure 4** illustrates the sequential development of SynComs to optimize plant–microbe interactions for enhanced crop production. The process starts by identifying bacterial strains with plant growth-promoting traits, such as indole acetic acid (IAA) production (Corkidi et al., 2012). The genetic variability of these strains is assessed for compatibility (Kumar and Pandey, 2013; Zhong et al., 2024), followed by a systematic evaluation of their effects on plant growth under controlled conditions, and validation through greenhouse and field trials. Finally, the SynComs are optimized for large-scale application, resulting in improved crop growth, enhanced nutrient uptake, and increased disease resistance (Blankinship et al., 2011). The figure magnifies the root system, highlighting the interactions between SynComs and the rhizosphere, emphasizing their role in nutrient cycling and plant health, thus contributing to sustainable agriculture (Guo et al., 2018; Boo et al., 2024).

Effectively utilizing the plant microbiome to enhance plant health, growth, and productivity involves several strategic approaches. First, selecting plant types with unique root exudate profiles is crucial, as this promotes beneficial microbial communities and enriches plant–microbe interactions by attracting advantageous microbial populations through selective breeding (Singh and Siddiqui, 2010; Blagodatskaya and Kuzyakov, 2018; Velez et al., 2018). Second, optimizing nutrient management is essential for maintaining appropriate nutrient levels to reduce stress-induced changes in exudate composition; optimal management lessens plant stress and preserves microbiome functions, preventing detrimental alterations in root exudate patterns (Corkidi et al., 2012; Singh and Jha, 2016; Xiong et al., 2019; Yang et al., 2024). Lastly, actively introducing beneficial microorganisms into the rhizosphere can enhance plant development and bolster resistance to stress and disease, thereby improving crop productivity and resilience, which are vital for sustainable agriculture (Strickland and Rousk, 2010).

#### 4.1.1 Manipulation of the rhizosphere microbiome to enhance plant growth and resilience

1. **Harnessing native microorganisms:** Select and transfer beneficial native microorganisms to harness the natural variety of bacteria associated with plants. This process enhances microbial capabilities and fosters robust mutualistic interactions. For example, crops like maize, rice, and sugarcane have host-specific bacterial clusters that contribute to the development of resilient microbiomes both inside the plants and in their surrounding environment (Elsayed et al., 2020; dos Santos et al., 2022; Medina and Kutuzova, 2022).
2. **Microbial engineering strategies:** Actively control the rhizosphere microbiome by identifying and utilizing core microbiome components to attract beneficial microbes. Cultivating plants with beneficial bacteria, integrating them into the plant’s existing microbial community, and using next-generation sequencing to identify the core microbiomes can refine microbial community composition. This approach also includes the application of microbial cocktails to seeds, ensuring the transmission of beneficial microbes to future generations (Fahad et al., 2015; Faust et al., 2015; Jha et al., 2018; Fabiańska et al., 2019; Franco-Duarte et al., 2019).

The effectiveness of manipulating the microbiome lies in understanding the intricate dynamics between plants and microbes. Tools like metagenomics, transcriptomics, and CRISPR-based techniques allow researchers to analyze these interactions. Metagenomics helps identify key microbial species and their roles in the rhizosphere, while omics technologies offer insights into gene expression and protein profiles that influence plant–microbe interactions. CRISPR can be used to edit plant genes, such as those influencing root exudate production, optimizing microbiome functions and plant performance (Zelezniak et al., 2015; MaChado et al., 2021; Chen et al., 2024).

#### 4.1.2 Biochemical approaches for enhancing plant–microbe interactions

Biochemical methods modify root exudates in response to environmental stressors. Plant-derived VOCs transmit signals to soil bacteria, helping to prevent diseases and enhance plant defense mechanisms. These VOCs can be utilized to selectively promote beneficial microorganisms and improve plant health (Gao et al., 2010; Ruan et al., 2022). Some techniques for enhancing plant growth and health through microbiome engineering are shown in **Table 4**, for example, *in situ* engineering methods replicate microbial quorum sensing, where bacteria communicate using signaling molecules to enhance microbiome development. This approach improves nutrient acquisition and provides protection against harmful chemicals by enhancing beneficial microbial interactions. Plant breeding techniques, such as quantitative trait locus (QTL) mapping, aid in selecting plant genotypes that support beneficial microbial communities, thereby promoting growth and health (Deng et al., 2014; Smith et al., 2019).

**Table 4:** Techniques for enhancing plant growth and health through microbiome engineering.

Microbiome	Techniques	Beneficial effects	References
Host mediated microbiome	Introduction of PGPR to plant	Enhances growth via nitrogen fixation and phosphorus solubilization	(Pierce et al., 2021)
Synthetic microbiome	Introduction of microbial consortia	Improves nutrient uptake and disease resistance	(Romdhane et al., 2022; Boo et al., 2024)
*In situ* microbiome	Manipulation of native microbial community	Modifies soil conditions, introduces specific microbial strains	(Rousk et al., 2010)
Phyto microbiome	Plant symbiont-based approach	Promotes growth of nitrogen-fixing bacteria or mycorrhizal fungi	(Foster and Bell, 2012)
Artificial seed microbiome	Introduction of PGPB to seeds	Enhances growth through nitrogen fixation, phosphorus solubilization	(Hibbing et al., 2010a)
Plant mycobiome	Optimization of fungal–plant interaction	Incorporates beneficial fungi, suppresses harmful fungi	(de Boer and van der Wal, 2018)
Rhizosphere microbiome	Bacterial competitiveness engineering	Outcompetes harmful bacteria, enhances beneficial bacteria	(Hardoim et al., 2015a)
Traditional microbiome	Agricultural management practices	Utilizes crop rotation, organic farming, and no-till agriculture	(Coyte et al., 2015)

PGPR, plant growth-promoting rhizobacteria; PGPB, plant growth-promoting bacteria.

### 4.2 Transgenic plants and the rhizosphere

Transgenic crops have the potential to enhance nutritional value and resistance to pests. However, concerns about their impact on soil microbial populations persist. These plants can improve nutrient absorption and environmental resilience but may alter root exudate composition compared to non-transgenic plants. As a result, the changes in exudates can persist in the soil, significantly affecting the native microbiota (Zengler and Zaramela, 2018). Studies on the effects of transgenic crops on rhizosphere biota remain limited, with varying results due to factors such as crop type and trial duration. Some studies suggest that transgenic crops may negatively affect native species through gene flow or the repeated use of insecticides and herbicides (Nazir et al., 2017). However, other studies show that genetically modified crops, like Bt maize, can increase biomass without notable adverse effects on soil microbiota. These conflicting findings highlight the need for further research to fully understand the long-term impact of transgenic crops on soil microorganisms (He et al., 2023). A deeper understanding of plant–microbe interactions and microbial community dynamics is essential for developing a robust and resilient rhizosphere microbiome. Additionally, optimizing techniques such as seed priming or encapsulation for introducing beneficial microorganisms is critical for ensuring their effectiveness in the soil (Du et al., 2022). These advancements will support sustainable agricultural practices that maximize the benefits of transgenic crops while minimizing environmental risks.

### 4.3 Formation of SynComs via exudate-mediated assembly

Root exudates, a diverse mix of organic chemicals produced by plant roots, play a crucial role in shaping the rhizosphere microbiome. These exudates attract and select specific microbes, facilitating the assembly of SynComs (Marín et al., 2021; Hu et al., 2022). Plants actively customize the composition of root exudates to recruit groups of microorganisms that provide distinct beneficial functions. For instance, carbohydrates such as glucose and fructose attract microbes adept at sugar metabolism, while organic acids like malic and citric acids draw in phosphate-solubilizing bacteria (Yuan et al., 2021). Exudates also influence competition among microorganisms, promoting the growth of beneficial species while inhibiting harmful ones (Wang and Kuzyakov, 2023). Certain exudates can suppress fungal pathogen proliferation, creating a favorable environment for beneficial bacteria. Additionally, exudates establish gradients in the rhizosphere, leading to the spatial organization of microbial communities, which enhances cooperation among diverse microbial groups (Wang and Kuzyakov, 2024b).

Once established, the composition of root exudates continues to influence SynCom members, enhancing their beneficial roles. Exudates supply essential nutrients, promoting the growth and activity of these microbial communities (Cornforth and Foster, 2013). Sugars and organic acids serve as carbon and energy sources, while amino acids provide nitrogen and other vital elements. Previous studies have indicated that exudates can boost the activity of SynCom members involved in nutrient cycling, such as nitrogen fixation and phosphate solubilization (Heilbronner et al., 2021). Specifically, exudates stimulate the expression of genes responsible for nitrogenase synthesis in nitrogen-fixing bacteria (Pantigoso et al., 2022a; Timofeeva et al., 2023). Furthermore, they can induce SynCom members to produce antimicrobial substances, thereby increasing their effectiveness against pathogens (Klimek et al., 2021; Ma et al., 2023). This ability to encourage beneficial bacteria to generate antibiotics and lytic enzymes highlights the critical role of root exudates in fostering synergistic relationships within the rhizosphere (Pausch and Kuzyakov, 2018).

The interaction between root exudates and SynComs is mutually beneficial. SynComs can alter the composition and release of root exudates, creating a cycle that promotes plant development and resilience (Bais et al., 2010). By metabolizing specific compounds or emitting their own secondary metabolites, SynCom members can modify root exudate composition, thereby changing the attractiveness of the rhizosphere to other microorganisms (Wang and Kuzyakov, 2024c). Moreover, SynComs can regulate exudate release by influencing plant signaling pathways. Beneficial bacteria produce signaling molecules that trigger the release of specific exudates, promoting their own growth and activity (Torres et al., 2005). This dynamic not only improves nutrient availability but also enhances plant growth, ultimately strengthening the plant’s resilience to environmental stresses and pathogen attacks (Hanson et al., 2018).

The development of SynComs in the rhizosphere is largely driven by root exudates, which are diverse organic compounds secreted by plant roots. These exudates serve as chemical signals to attract and selectively recruit beneficial microbes, facilitating the assembly of SynComs. This dynamic interaction is essential for strengthening plant–microbe relationships and supporting plant health and productivity (Wang et al., 2021). Soil-dwelling bacteria possess the ability to sense environmental signals and communicate through signaling molecules. This microbial signaling is pivotal in recruiting beneficial taxa, often drawn to specific root exudates. The selective recruitment of these microbes enhances the establishment of SynComs, promoting plant resilience and adaptability to environmental stresses (Kästner et al., 2021). Once recruited, beneficial microbes form direct interactions with plant roots, triggering physiological changes or activating stress responses. These interactions enable a symbiotic exchange, which improves nutrient uptake, disease resistance, and overall growth. The signaling molecules produced by microbes can influence plant signaling pathways, amplifying the benefits of these partnerships (LaRowe and Van Cappellen, 2011; Maurice et al., 2024).

**Figure 5:**
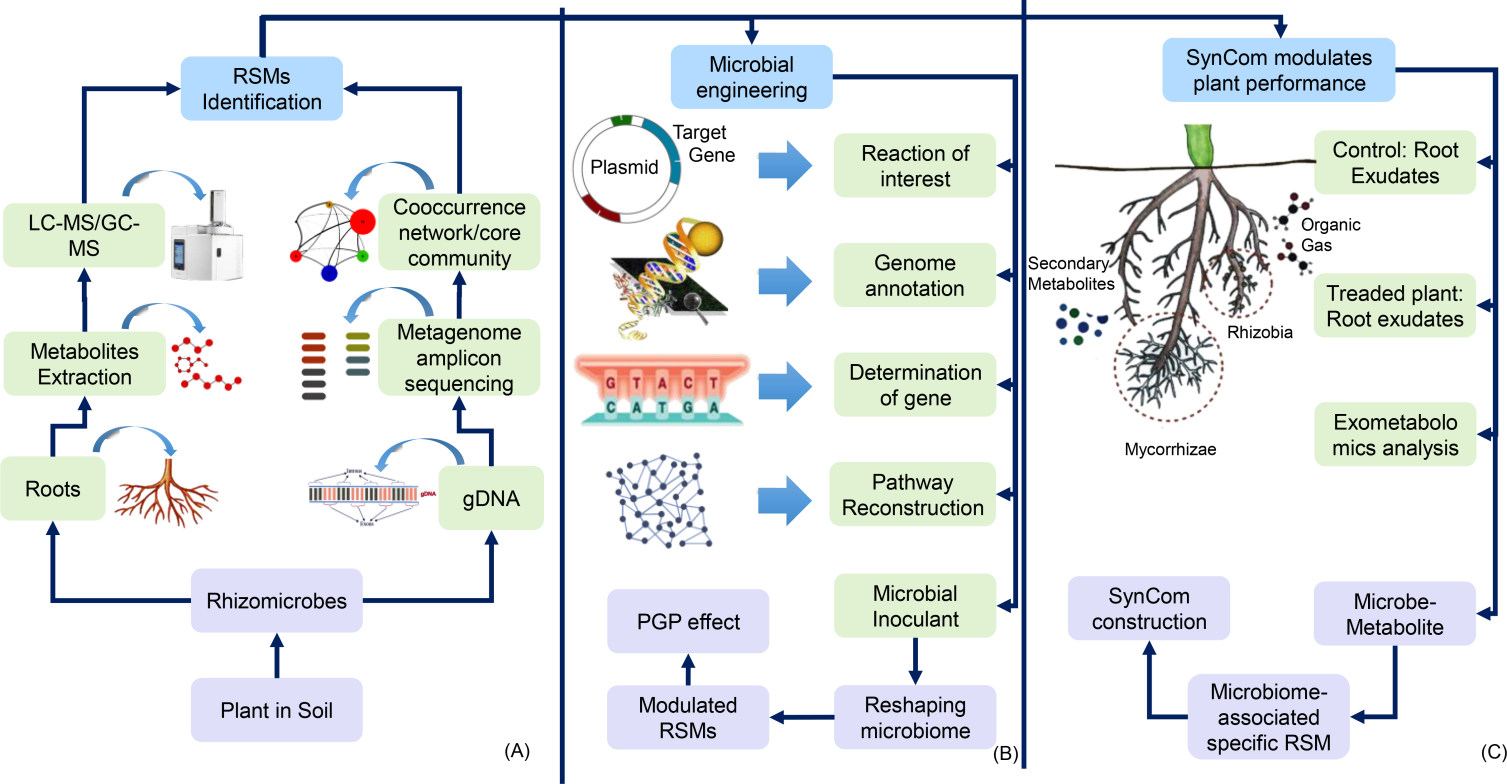
Strategies for microbial engineering and synthetic microbial community (SynCom) optimization. This figure outlines the methodologies for identifying root-associated microbes (RSMs) and engineering SynComs to enhance plant performance. **(A)** illustrates the identification processes using techniques like liquid chromatography–mass spectrometry (LC–MS) and metagenomic sequencing. **(B)** details the microbial engineering approaches, including genome annotation and pathway reconstruction. **(C)** demonstrates how engineered SynComs can modulate plant performance by controlling root exudates and reshaping microbial communities, ultimately improving plant growth and resilience.

Advances in microbial engineering techniques are depicted (**Figure 5**) to improve the identification and optimization of root-associated microbial systems (RMS). Techniques such as liquid chromatography–mass spectrometry (LC–MS) and co-occurrence network analysis are instrumental in characterizing beneficial microbial taxa and their interactions (Gunina and Kuzyakov, 2022). These tools enable researchers to reconstruct tailored microbial communities that enhance nutrient cycling, stress tolerance, and overall plant performance (Gunina et al., 2017 ; Díaz-Vallejo et al., 2021). Engineered SynComs provide a promising strategy for optimizing plant health and agricultural productivity. By fine-tuning microbial interactions and improving the functionality of specific strains, these designed communities significantly bolster plant resilience against abiotic and biotic stresses, thereby promoting sustainable agricultural practices (Heilbronner et al., 2021; Sabrina et al., 2021; Al-Khayri et al., 2023). Therefore, exudate-mediated SynCom formation represents a dynamic, mutually beneficial process that enhances both plant and microbial health. Understanding the complex interactions and signaling mechanisms involved in this process offers valuable insights for developing innovative strategies to make plants healthy and more stress tolerant (Klimek et al., 2021). These advancements hold immense potential for fostering sustainable agriculture and ensuring global food security (Klimek et al., 2021).

### 4.4 Selective breeding for enhanced microbial interactions

Selective breeding of plants to improve their interactions with microorganisms has become increasingly important. This process can lead to the selection of microbes that engage in beneficial interactions with plant roots and surrounding soil. For example, genes responsible for root structure and exudation can influence microbial communities in the rhizosphere (Tijhuis et al., 1993; Haney, 2021a). Mutations affecting root hair size and density can reduce microbial diversity, indicating that specific root characteristics impact the recruitment of beneficial microbes (Guhr et al., 2015). Studies involving maize have identified genetic loci that support the optimization of symbiotic relationships between plants and microbes (Wolf et al., 2013; Fan et al., 2017; Rivas-Franco et al., 2020). For instance, breeding crops to improve compatibility with symbiotic microorganisms, such as *Epichloë* endophytes or AMF, enhances their effectiveness. In ryegrass, selective breeding for endophyte alkaloid profiles has led to improved pest resistance and consistent trait expression (Calvin and Bassham, 1962; Rojas-Solís et al., 2018; Schimel, 2018; Zheng et al., 2018; Rolfe et al., 2019; Sharma et al., 2020; Wang and Kuzyakov, 2024b). Therefore, the goal is to enhance plant resilience against abiotic stress and improve nutritional quality. There is increasing interest in using plant-associated microbes to enhance plants’ ability to tolerate stress while reducing pesticide residues. Soil- and plant-associated microbes can degrade pesticides, thus improving the safety of plant products (Brunel et al., 2020; Dubey et al., 2020). Additionally, endophytes can help break down herbicides, providing a mechanism for herbicide resistance and effective weed management (Lemanceau et al., 2017; Deveau et al., 2018; Jacoby et al., 2020b; Compant et al., 2021; Pantigoso et al., 2022b; Wang Y. et al., 2024).

### 4.5 Advanced experimental techniques for understanding microbial community diversity and plant–microbe interactions

Advanced experimental techniques are essential for understanding microbial community diversity and plant–microbe interactions. Functional gene microarrays, such as PhytoChip and GeoChip, allow researchers to assess microbial processes and gain insights into the functional roles of microbial taxa within an ecosystem (Decleyre et al., 2015; Meidute et al., 2018). Real-time visualization of microbial colonization on plant roots is possible through fluorescence emission *in situ*, offering valuable data on microbial interactions in natural environments (Hardoim et al., 2015b; Sayre et al., 2023; Haney, 2021b). Molecular manipulation tools, such as gene silencing, transgenic technologies, and CRISPR/Cas9 gene editing, have become integral for studying plant–microbe interactions. These technologies allow for the investigation of genetic functions and the refinement of traits that benefit plant performance. CRISPR/Cas9, known for its precision, can be used to modify plant genes affecting root exudates and microbiome composition, thereby improving plant growth and resilience (Lo et al., 2022; Chen et al., 2024).

Moreover, synthetic biology and genetic engineering provide solutions to agricultural challenges, including pest resistance, nutrient efficiency, and environmental resilience. The application of these techniques holds the potential to enhance agricultural sustainability, increase crop yields, and address global food security concerns (Santín et al., 2016; Aplakidou et al., 2024). RNA interference (RNAi) is also an effective tool for improving disease resistance by targeting both transcriptional and posttranscriptional processes (Mason-Jones et al., 2022; Wang M. et al., 2023).

**Figure 6:**
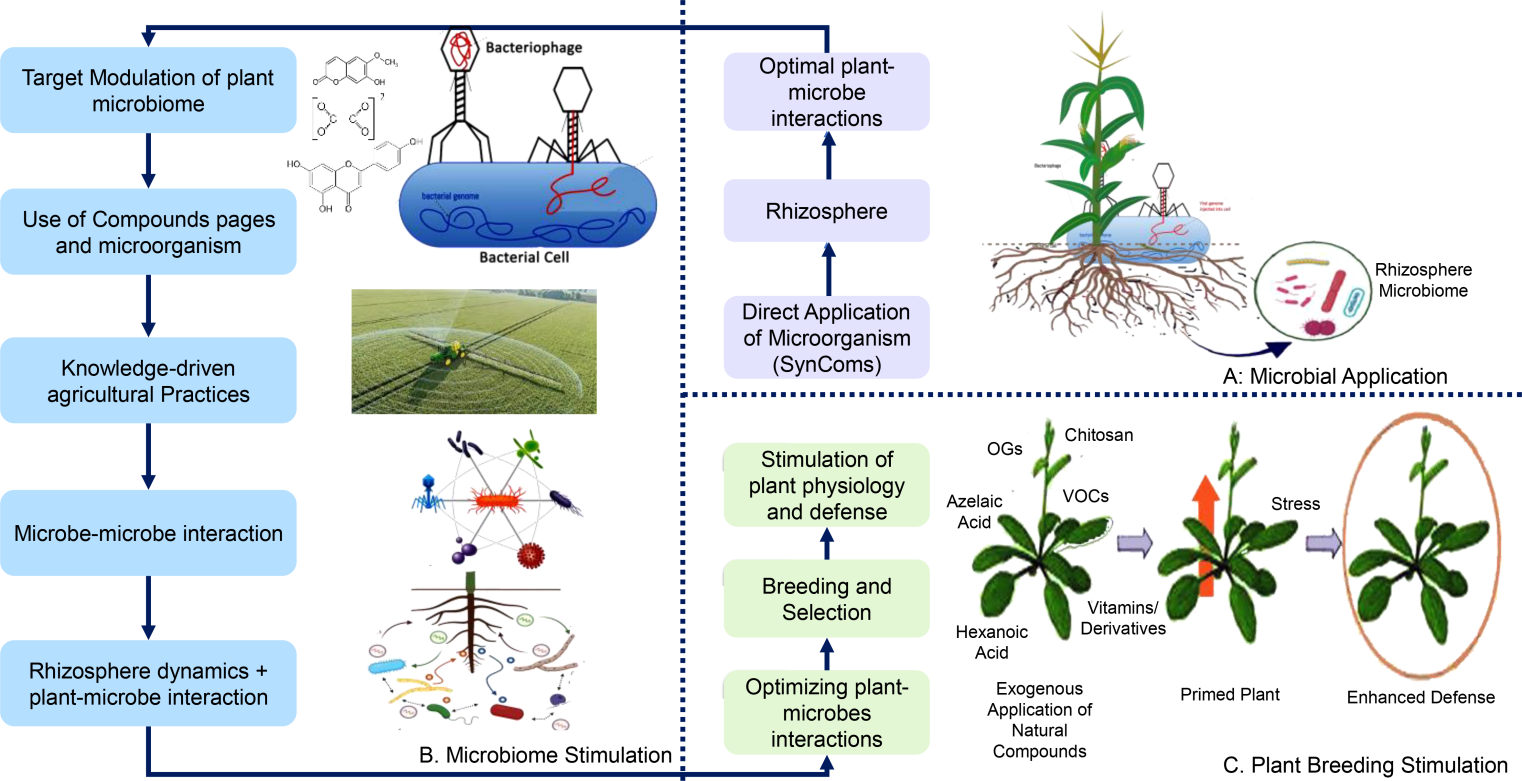
Strategies for modulating plant microbiomes. This figure presents an overview of strategies for optimizing plant–microbe interactions to enhance agricultural practices. **(A)** outlines the targeted modulation of the plant microbiome, including the use of specific compounds and knowledge-driven approaches. **(B)** emphasizes microbiome stimulation through direct applications of synthetic microbial communities (SynComs) to improve rhizosphere dynamics. **(C)** highlights plant breeding strategies aimed at enhancing plant performance through optimized plant–microbe interactions, showcasing how these methods can lead to improved crop resilience and yield.

**Figure 7:**
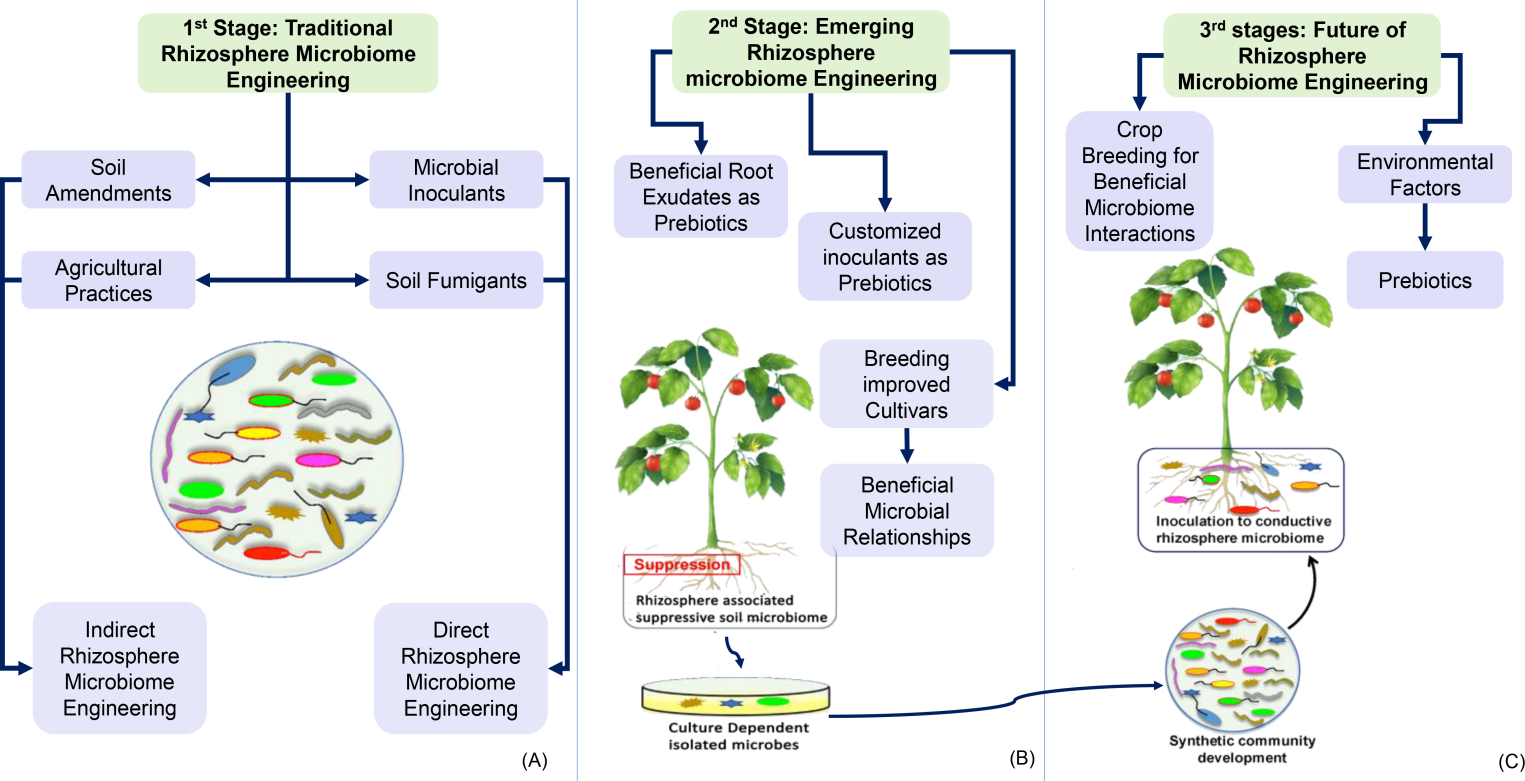
Rhizosphere microbiome engineering for sustainable agriculture. This figure illustrates the three stages of rhizosphere microbiome engineering aimed at enhancing plant–microbe interactions for sustainable agricultural practices. **(A)** First stage: traditional rhizosphere microbiome engineering. This stage focuses on techniques such as microbial inoculants and soil amendments that enhance soil health and support beneficial microbial communities. **(B)** Second stage: emerging rhizosphere microbiome engineering. This stage emphasizes breeding plants for beneficial root exudate production and developing customized microbial inoculants to strengthen plant–microbe relationships. **(C)** Third stage: crop breeding for beneficial microbiome interactions. This final stage highlights the importance of selecting crop varieties that promote beneficial microbial communities and environmental factors that support these interactions.

“Rhizosphere microbiome engineering” (**Figures 6**, **7**) depicts three stages of enhancing plant–microbe interactions to promote sustainable agriculture. In **Figure 6**, the first stage focuses on traditional methods, such as soil management and microbial inoculants, to boost beneficial microbial populations. The second stage introduces emerging techniques that involve breeding plants for beneficial root exudate production and developing customized inoculants (Singer et al., 2016). The third stage emphasizes the role of genetic engineering and molecular methods, showcasing how these advanced techniques can be used to tailor plant traits for improved interactions with beneficial microbes, thereby enhancing nutrient acquisition, stress tolerance, and overall plant health. This integration of genetic engineering with microbiome strategies represents a progressive approach to achieving resilient agricultural systems (Rojas-Solís et al., 2018; Bai et al., 2023).

**Figure 8:**
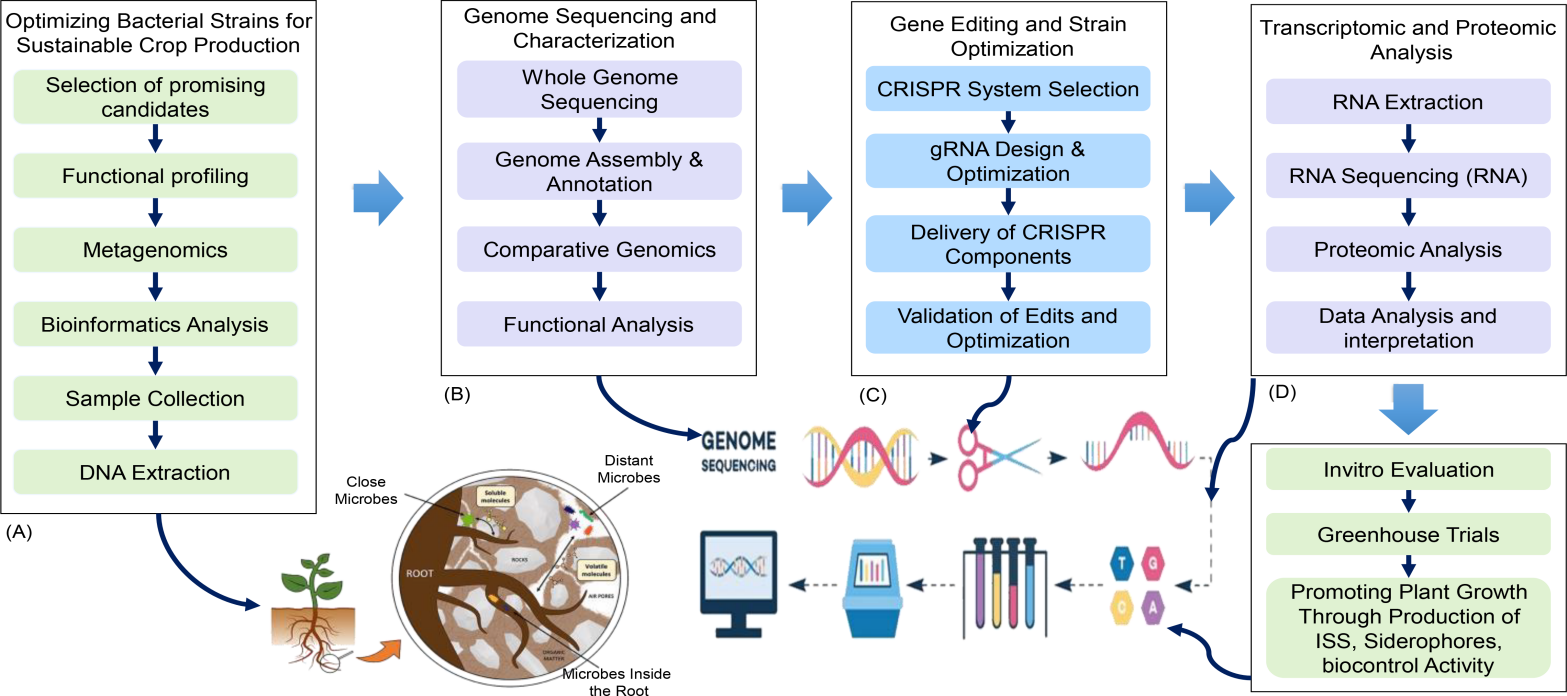
Strategies for sustainable crop production through bacterial strain utilization: outlines methodologies for selecting and optimizing bacterial strains to enhance sustainable crop production using genomic and molecular techniques. The figure presents four key stages: **(A)** isolating bacterial strains through functional profiling and metagenomics, **(B)** utilizing whole-genome sequencing for genome assembly and annotation, **(C)** employing CRISPR technology for gene editing and optimization, and **(D)** conducting transcriptomic and proteomic analyses to evaluate gene expression and protein functions. This integrated approach highlights the potential of modern techniques to improve nutrient uptake, promote plant growth, and provide biocontrol, advancing sustainable agriculture through the effective use of beneficial microbes.

As depicted in **Figure 8**, methodologies for optimizing bacterial strains to enhance sustainable agriculture are essential for improving crop production systems. The process begins with the isolation of promising bacterial strains through functional profiling, metagenomics, and biochemical analysis (Berendsen et al., 2012; Bulgarelli et al., 2013; Müller et al., 2016; Zhang et al., 2020; Solomon and Janda, 2024b). Following the isolation, genome sequencing is conducted to annotate and identify beneficial traits associated with plant growth promotion and stress resilience (Hacquard et al., 2015). Subsequently, the application of CRISPR gene editing allows for the refinement of these beneficial traits, ensuring that the selected strains can effectively support plant growth and enhance resilience to environmental stresses (Calvin and Bassham, 1962; Geldner, 2013; Carabotti et al., 2015; Berendsen et al., 2018; Cryan et al., 2019; Hulme et al., 2020). Finally, transcriptomic and proteomic analyses are employed to evaluate gene expression and protein functions, guiding the development of bacterial strains that improve nutrient uptake and provide biocontrol against pathogens (Yuan et al., 2018). This integrated approach underscores the potential of modern genomic techniques in leveraging beneficial microbes for sustainable crop production, offering innovative solutions to meet the challenges of food security and environmental sustainability (Bais et al., 2010; Hiruma et al., 2016; Bakker et al., 2018; Stringlis et al., 2018b; Finkel et al., 2019).

## 5 Current knowledge gaps and future directions

### 5.1 The importance of the rhizosphere ecosystem

The rhizosphere, the soil region surrounding plant roots, represents a dynamic ecosystem where complex interactions between root exudates, SynComs, and diverse soil microorganisms shape plant health and nutrient availability. These interactions are largely mediated by root exudates, which vary in composition and concentration based on plant species, developmental stages, and environmental conditions (Brunel et al., 2020; Pascale et al., 2020). However, isolating the specific impacts of individual exudate components on SynCom members is challenging, given the competition among microbial species for limited resources. This complexity underscores the need for a more comprehensive understanding of the genetic and metabolic mechanisms underlying these interactions (Mark et al., 2005; DeAngelis et al., 2009; Micallef et al., 2009; Chaparro et al., 2013).

Despite significant advancements in microbial ecology, the application of SynComs in agricultural settings presents challenges, including regulating colonization, maintaining functional stability, and ensuring competitiveness against native microbes (Cavaglieri et al., 2009). Furthermore, spatial and temporal variability in management practices, such as tillage and fertilization, often affects the effectiveness of these microbial applications (Prando et al., 2024). Therefore, advancing our understanding of the mechanisms driving root colonization and microbial functionality is critical for achieving success in rhizosphere engineering.

#### 5.1.1 Development and application of SynComs

To enhance the stability and efficacy of SynComs in diverse agricultural environments, research should prioritize their sustainable production and integration into existing practices (Lundberg et al., 2012). For instance, incorporating SynComs into systems that involve tillage, irrigation, and organic amendments, such as compost or manure, could promote microbial diversity and resilience (Bulgarelli et al., 2012; Lemanceau et al., 2017). Synthetic biology also presents opportunities for engineering rhizobacteria with specific traits to enhance nutrient mobilization and plant health (Jacoby et al., 2020b).

Although challenges remain, SynComs offers immense benefits for sustainable agriculture. These include improved nutrient cycling, reduced reliance on synthetic fertilizers and pesticides, and increased plant resilience to abiotic stresses such as drought and salinity (Dennis et al., 2010; Dubey et al., 2020). However, inconsistencies in experimental design and terminology can hinder reproducibility and data comparability. Standardized frameworks emphasizing clear methodologies and rigorous evaluation are essential to overcome these obstacles (Hardoim et al., 2015b).

### 5.2 Methodological considerations for SynCom research

Effective SynCom research requires careful consideration of experimental design, plant models, and environmental variables. Selecting well-established plant models can provide insights into microbiome dynamics and their influence on plant development. Additionally, external factors, such as soil composition and climate, should be incorporated into research frameworks to understand how these variables affect plant–microbe interactions (Stursova et al., 2012; Sheth et al., 2016; Seidel et al., 2024). Therefore, advancements in omics technologies metagenomics, metatranscriptomics, and metabolomics are transforming our understanding of microbial community dynamics in the rhizosphere. Metagenomics allows for the identification of genetic potential within microbial communities, while metatranscriptomics reveals active microbial functions, shedding light on their roles in nutrient exchange and plant health (Mackelprang et al., 2016; Haney, 2021b; Wang Y. et al., 2023). These insights can guide the development of more targeted microbial interventions for sustainable agriculture.

### 5.3 Advances in “omics” approaches

Omics technologies have revolutionized the study of rhizosphere dynamics. Metabolomics, for example, provides insights into small-molecule metabolites involved in plant–microbe symbiosis, which can inform strategies for improving plant resilience and optimizing agricultural practices (Bais et al., 2010; Lo et al., 2022). Integrating data from genomics, transcriptomics, metagenomics, and metabolomics facilitates a holistic understanding of microbial ecosystems, revealing regulatory networks and key metabolic pathways critical for plant–microbe interactions (Aplakidou et al., 2024). For instance, variations in root exudate levels correlate with specific microbial groups, emphasizing the importance of harnessing these chemical signals to enhance microbiome functionality (Tong et al., 2024). Despite these advancements, challenges remain, particularly in integrating data across molecular levels. Addressing these computational hurdles is essential to fully leverage the potential of multi-omics technologies in understanding and optimizing rhizosphere interactions.

## 6 Conclusion

The engineering of root exudates emerges as a cornerstone for designing resilient rhizosphere microbiomes, essential for advancing plant health and sustainable agriculture. Root exudates, composed of diverse compounds such as organic acids, amino acids, sugars, and secondary metabolites, serve as key biochemical signals that influence the composition, behavior, and functionality of microbial communities in the rhizosphere. These interactions underpin critical processes like nutrient cycling, pathogen suppression, and plant adaptation to environmental stresses, making them indispensable for maintaining agricultural productivity and ecological balance. Optimizing root exudates to align with plant-specific and environmental requirements holds significant potential for enhancing the effectiveness of synthetic microbial communities. Tailored SynComs can amplify plant resilience to abiotic stresses, such as drought and salinity, while simultaneously mitigating biotic stresses like pests and diseases. Future research must prioritize unraveling the molecular mechanisms underlying root exudate-mediated microbial interactions, the role of environmental factors in shaping exudate composition, and the specific needs of diverse crop species under varying agroecological conditions. The integration of insights from microbiology, plant sciences, and environmental science research will be crucial for engineering root exudates to create resilient and productive agricultural systems. Such a multidisciplinary approach can facilitate the design of microbial consortia that not only boost crop yields and reduce reliance on synthetic inputs but also safeguard long-term soil health and biodiversity. By harnessing the functional potential of the rhizosphere microbiome and the signaling properties of root exudates, we can achieve transformative agricultural solutions that address global food security challenges (Sustainable Development Goal). Ultimately, this strategy offers a path toward sustainable farming practices that balance productivity with environmental preservation, ensuring resilient agricultural landscapes capable of adapting to an evolving climate and growing population demands.

## Data Availability

The original contributions presented in the study are included in the article/supplementary material. Further inquiries can be directed to the corresponding authors.
